# Supplementary material for: Whole Blood Proteome Dynamics Defines Predictive Diagnostic and Prognostic Signatures of Cryptococcal Infection
Source: Mol Cell Proteomics. 2025 Oct 7;24(11):101083. doi: 10.1016/j.mcpro.2025.101083 (PMC12664494; doi:10.1016/j.mcpro.2025.101083)
Supplement: SupplementaryTable S1-S11 [file mmc1.docx]

**Supplemental Table 1: Whole blood and cardiac blood experimental and gas phase fractionation liquid chromatography gradient and configuration.**

| Blood Sample Gradient Specifications and LC Configuration | | | |
| --- | --- | --- | --- |
| Gradient | Time (min) | % Mobile Phase B | Flow (μl/min) |
|  | 0 | 10 | 2.0 |
|  | 0.3 | 10 | 2.0 |
|  | 0.6 | 10 | 0.8 |
|  | 13.6 | 22.5 | 0.8 |
|  | 20.5 | 35.0 | 0.8 |
|  | 20.9 | 55.0 | 2.0 |
|  | 20.95 | 99.0 | 2.0 |
|  | 22.35 | 99.0 | 2.0 |
| LC Parameters | LC Configuration | Trap and Elute | |
|  | Fast Loading/Equilibration Mode | Pressure Control | |
|  | Loading/Equilibration/Wash Pressure | Max Pressure | |
|  | Equilibration Factor | 3 | |
|  | Sampler Temperature | 7 °C | |
|  | Mobile Phase A / Weak Wash | 0.1% Formic Acid in Water | |
|  | Mobile Phase B / Strong Wash | 0.1% Formic Acid in 80% Acetonitrile | |
|  | Zebra Wash | Enabled | |
|  | Zebra Wash Cycles | 4 | |
|  | Analytical Column Temperature | 50 °C | |
| Column Specifications | Analytical Column | EASY-Spray™ PepMap™ Column, 2µm C18, 150µm × 15 cm (P/N ES906) | |
|  | Trap Column | PepMap™ Neo Trap Cartridge, 5 μm C18 300 μm x 5 mm, (P/N 174500) | |

**Supplemental Table 2. Orbitrap Astral Zoom mass spectrometer global source and mass spectrometer parameters.**

| Global Parameters (Source & MS) | |
| --- | --- |
| Positive Ion Voltage | 2100 Volts |
| Ion Transfer Tube Temperature | 290 °C |
| Expected Peak Width | 10 seconds |
| Default Charge State | 2 |
| Lock Mass Correction | Off |

**Supplemental Table 3. Orbitrap Astral Zoom mass spectrometer MS1 full scan experiment parameters.**

| MS1 Full Scan Experiment Parameters | |
| --- | --- |
| Orbitrap Resolution | 240K |
| Scan Range (*m/z*) | 380-980 |
| RF Lens (%) | 40 |
| Normalized AGC Target (%) / Absolute AGC Value | 500% / 5.00e6 |
| Maximum Injection Time | 5 milliseconds |
| Microscans | 1 |

**Supplemental Table 4. Orbitrap Astral Zoom mass spectrometer MS2 DIA scan experiment parameters.**

| MS2 DIA Scan Experiment Parameters | |
| --- | --- |
| Precursor Mass Range (*m/z*) | 380-980 |
| Isolation Window (*m/z*) | 3 |
| Window Placement Optimization | On |
| AGC Target | Custom |
| Normalized AGC Target (%) / Absolute AGC Value | 500% / 5.00e4 |
| Maximum Injection Time | 7 milliseconds |
| DIA Scan Range (*m/z*) | 150-2000 |
| HCD Collision Energy (%) | 25 |
| RF Lens (%) | 40 |
| Pre-Accumulation | On |
| Loop Control | Time |
| Time | 0.6 seconds |

**Supplemental Table 5. Orbitrap Astral Zoom mass spectrometer gas phase fractionation MS1 full scan experiment parameters.**

| GPF MS1 Full Scan Experiment Parameters | |
| --- | --- |
| Orbitrap Resolution | 240K |
| Scan Range (*m/z*) | Incremental 100 *m/z* scans (380-480; 480-580; 580-680; 680-780; 780-880; 880-980) |
| RF Lens (%) | 40 |
| Normalized AGC Target (%) / Absolute AGC Value | 500% / 5.00e6 |
| Maximum Injection Time | 3 milliseconds |
| Microscans | 1 |

**Supplemental Table 6. Orbitrap Astral Zoom mass spectrometer gas phase fractionation MS2 DIA scan experiment parameters.**

| GPF MS2 DIA Scan Experiment Parameters | |
| --- | --- |
| Precursor Mass Range (*m/z*) | Incremental 100 *m/z* scans (380-480; 480-580; 580-680; 680-780; 780-880; 880-980) |
| Isolation Window (*m/z*) | 1 |
| Window Placement Optimization | On |
| AGC Target | Custom |
| Normalized AGC Target (%) / Absolute AGC Value | 500% / 5.00e4 |
| Maximum Injection Time | 18 milliseconds |
| DIA Scan Range (*m/z*) | 150-2000 |
| HCD Collision Energy (%) | 25 |
| RF Lens (%) | 40 |
| Pre-Accumulation | On |
| Loop Control | Time |
| Time | 0.6 seconds |

**Supplemental Table 7. *Cryptococcus neoformans* gas phase fractionation liquid chromatography gradient and configuration.**

| *C. neoformans* Gradient Specifications and LC Configuration | | | |  |  |
| --- | --- | --- | --- | --- | --- |
| Gradient | | Time (min) | % Mobile Phase B | Flow (μl/min) | |
|  |  | 0 | 8 | 0.5 | |
|  |  | 2.5 | 8 | 0.5 | |
|  |  | 3.0 | 8 | 0.25 | |
|  |  | 37.0 | 22.5 | 0.25 | |
|  |  | 48.5 | 35 | 0.25 | |
|  |  | 48.9 | 98 | 0.25 | |
|  |  | 49.0 | 98 | 0.5 | |
|  |  | 54.0 | 98 | 0.5 | |
| LC Parameters | | LC Configuration | Trap and Elute | | |
|  |  | Fast Loading/Equilibration Mode | Pressure Control | | |
|  |  | Loading/Equilibration/Wash Pressure | Max Pressure | | |
|  |  | Equilibration Factor | 3 | | |
|  |  | Sampler Temperature | 7°C | | |
|  |  | Mobile Phase A / Weak Wash | 0.1% Formic Acid in Water | | |
|  |  | Mobile Phase B / Strong Wash | 0.1% Formic Acid in 80% Acetonitrile | | |
|  |  | Zebra Wash | Enabled | | |
|  |  | Zebra Wash Cycles | 4 | | |
|  |  | Analytical Column Temperature | 50°C | | |
| Column Specifications | | Analytical Column | EASY-Spray™ PepMap™ Neo Column, 2µm C18, 75µm × 50 cm (P/N ES75500PN) | | |
|  |  | Trap Column | PepMap™ Neo Trap Cartridge, 5 μm C18 300 μm x 5 mm, (P/N 174500) | | |

**Supplemental Table 8. Pulsar search results for whole blood and cardiac blood study specific spectral library.**

| Whole Blood and Cardiac Blood Study Specific Pulsar Search Results | |
| --- | --- |
| Number of Samples | 68 |
| Precursors | 51,193 of 51,214 |
| Modified Peptides | 41,173 of 41,184 |
| Peptides | 37,166 of 37,171 |
| Proteotypic Peptides | 34,846 |
| Protein Groups | 4017 (4016) |
| Proteins | 4158 (4157) |
| Single Hits | 1087 (1,199) |
| Fragments | 306,662 of 1,412,229 |
| Software Version | 19.8.250311.62635 |
| Merged | False |
| Search Engine Platform | Pulsar |

**Supplemental Table 9. Pulsar search results for whole blood and cardiac blood gas phase fractionation spectral library.**

| Whole Blood and Cardiac Blood Gas Phase Fractionation Pulsar Search Results | |
| --- | --- |
| Number of Samples | 24 |
| Precursors | 41,986 of 41,999 |
| Modified Peptides | 36,718 of 36,725 |
| Peptides | 33,649 of 33,652 |
| Proteotypic Peptides | 31,508 |
| Protein Groups | 4274 (4274) |
| Proteins | 4401 (4401) |
| Single Hits | 1152 (1244) |
| Fragments | 251,523 of 1,097,983 |
| Software Version | 19.8.250311.62635 |
| Merged | False |
| Search Engine Platform | Pulsar |

**Supplemental Table 10. Pulsar search results for *Cryptococcus neoformans* gas phase fractionation spectral library.**

| *Cryptococcus neoformans* Gas Phase Fractionation Pulsar Search Results | |
| --- | --- |
| Number of Samples | 6 |
| Precursors | 105,100 of 105,205 |
| Modified Peptides | 93,831 of 93,871 |
| Peptides | 85,824 of 85,839 |
| Proteotypic Peptides | 81,539 |
| Protein Groups | 4274 (4274) |
| Proteins | 5884 (5884) |
| Single Hits | 366 (430) |
| Fragments | 629,041 of 2,748,792 |
| Software Version | 19.8.250311.62635 |
| Merged | False |
| Search Engine Platform | Pulsar |

**Supplemental Table 11. Combined whole blood and cardiac blood study specific spectral library, blood and cardiac blood gas phase fractionation, and *Cryptococcus neoformans* gas phase fractionation spectral library composition.**

| Cryptococcus neoformans Gas Phase Fractionation Pulsar Search Results | |
| --- | --- |
| Number of Samples | 98 |
| Precursors | 161,955 of 161,955 |
| Modified Peptides | 140,321 of 140,321 |
| Peptides | 127,822 of 127,822 |
| Proteotypic Peptides | 120,721 |
| Protein Groups | 10,590 (10,637) |
| Proteins | 10,907 (10,953) |
| Single Hits | 1642 (1674) |
| Fragments | 969,614 of 4,262,012 |
| Software Version | 19.8.250311.62635 |
| Merged | True |
| Search Engine Platform | Pulsar |
